# Interferometric Laser Scanner for Direction Determination

**DOI:** 10.3390/s16010130

**Published:** 2016-01-21

**Authors:** Gennady Kaloshin, Igor Lukin

**Affiliations:** V.E. Zuev Institute of Atmospheric Optics SB RAS, Academician Zuev Square 1, Tomsk 634055, Russia; lukin_ip@iao.ru

**Keywords:** atmospheric turbulence, laser scanning, interference, laser beam

## Abstract

In this paper, we explore the potential capabilities of new laser scanning-based method for direction determination. The method for fully coherent beams is extended to the case when interference pattern is produced in the turbulent atmosphere by two partially coherent sources. The performed theoretical analysis identified the conditions under which stable pattern may form on extended paths of 0.5–10 km in length. We describe a method for selecting laser scanner parameters, ensuring the necessary operability range in the atmosphere for any possible turbulence characteristics. The method is based on analysis of the mean intensity of interference pattern, formed by two partially coherent sources of optical radiation. Visibility of interference pattern is estimated as a function of propagation pathlength, structure parameter of atmospheric turbulence, and spacing of radiation sources, producing the interference pattern. It is shown that, when atmospheric turbulences are moderately strong, the contrast of interference pattern of laser scanner may ensure its applicability at ranges up to 10 km.

## 1. Introduction

In a previous paper [1], we developed a method according to which, for a synchronous scanning with two laser beams, a wave interference forms in the region of their superposition, with the frequency of resulting oscillation being uniquely related to the direction toward the source. As is well known, the possibility of recording the contrast of interference pattern, formed by optical waves in the atmosphere, is determined both by coherence of the sources of optical radiation, and by random inhomogeneities of refractive index of air along the radiation propagation path [2,3]. In this paper, the method, developed for fully coherent beams, is generalized for the case when interference pattern is formed in the turbulent atmosphere by two partially coherent sources. The analysis performed identified the conditions under which stable interference pattern can be formed in the turbulent atmosphere on extended paths 0.5–10 km in length. In the paper, we describe in detail the method for selecting instrument parameters, ensuring the necessary instrument operability ranges in the atmosphere for any turbulence characteristics possible. The method is based on analysis of the mean intensity of interference pattern, produced by two partially coherent sources of optical radiation. For the chosen instrument parameters of interferometric laser scanning (ILS), we presented the cross sections of distribution of the mean intensity of interference pattern, produced by ILS in the turbulent atmosphere. Visibility of this interference pattern is estimated as a function of propagation pathlength and structure parameter of atmospheric turbulence for a few spacings of radiation sources, producing the interference pattern. ILS may be applied in navigation for determine the direction and the control of changing transient processes in the turbulent atmosphere. In particular, it may be valuable as a laser-sensing instrument in atmospheric optics for the study of intensity fluctuations during the propagation of optical radiation and as applied to an optical refraction, and investigation of a mirage detection.

## 2. Method

### 2.1. ILS for Direction Specification

**Figure 1:**
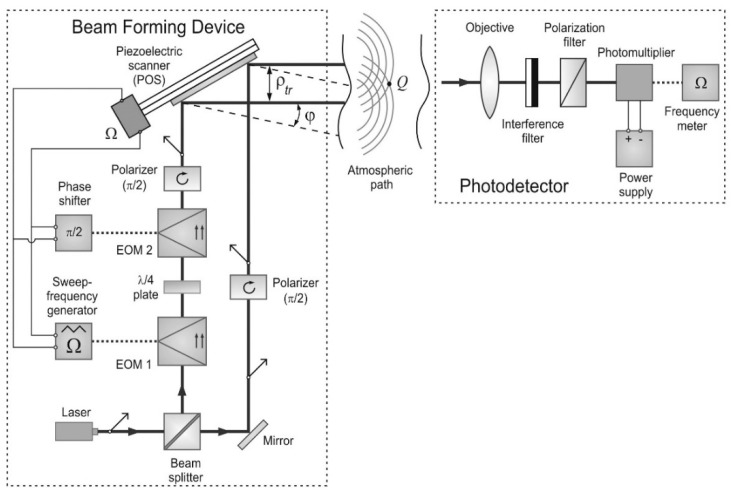
The optical scheme of interferometric laser scanner for direction specification.

Figure 1 shows the optical scheme of ILS, implementing this method for one-dimensional case. Using beam splitter and mirror, laser beam with polarization along *OX* axis is split into two parallel beams with identical apertures. One of the beams, after being reflected by mirror, passes through a polarizer, which turns the polarization plane of the beam through 90°, and arrives at piezoelectric scanner (PS). The second beam passes through electro-optic modulator (EOM) 1 operating on the basis of longitudinal electro-optic effect, through λ/4 plate (quarter-wave plate), and the second (analogous to the first) electro-optic modulator. Then, the second beam passes through the polarizer, which transmits radiation with polarization along the *OY* axis, and also arrives at the PS mirror. Sawtooth voltage from sweep-frequency generator (SFG) with frequency Ω is applied to EOM. This voltage is applied to EOM 1 directly and to EOM 2 via π/2 phase shifter; thus, the voltages at EOM 1 and EOM 2 have the phase difference of π/2.

A radiative component with polarization along the *OY* axis is frequency shifted by amount Ω relative to the initial laser beam. Thus, two parallel laser beams with identical polarizations arrive at PS. PS represents a mirror, mounted on piezoceramic plate. The principle of the work of PS is based on the use of reverse piezoelectric effect that can be identified in the deformation of crystal placed into the electric field at the certain orientation of lines of force of the field and direction of electric axis of the crystal analogously to the method of a beam forming device, as described in previous work [1]. When control voltage is applied to PS, the parallel laser beams scan in the plane, perpendicular to the plane, containing the optical axes of these beams. The control voltage for feeding PS is applied directly from SFG, which feeds EOM. Then, the scan angle ϕ is directly proportional to the frequency Ω. Because of diffraction divergence, the beams will start to overlap at a certain range from PS, where there will be an interference pattern with amplitude modulation of radiation with frequency Ω.

The optical signal of POS is accepted by photodetector device (PD) on moving object, consisting of objective, interference and polarization filters, and photodetector with power supply and frequency meter, which measures the frequency Ω. The onboard photoreceiver device records and extracts the frequency of the recorded signal Ω, which is uniquely related to the direction toward PS. The positions of the laser beams at the point of space Q (Figure 1) are determined by the PS statement controlled with electrical signal of Ω frequency. By means of optical amplification, the change of light beam direction to 1 s of arc is accessible. Hence, setting the direction to within 1 s of the arc is not technically challenging. As such, the accuracy of measurements may exceed at least 1/5000 s of the arc. This unveils wide opportunities in application of the method in both the research and engineering. In particular, it may be valuable in optical refraction spectroscopy, in photothermal deflection spectroscopy for controlling the slowly changing transient processes in gaseous and liquid media and plasma, in atmospheric optics for studying the intensity fluctuations during the propagation of optical radiation, in investigation of mirage detection and reflectance techniques, in geodesy, optical profilometry, ranging, and navigational course detection. At present, this method is used for direction control at high accuracy, with the capabilities of the techniques being limited by the sizes of laser beams. The method of beam forming device (BFD) has essentially the best capabilities in terms of resolution, because the resolution is determined by interference bandwidth.

### 2.2. Distortions of ILS-Formed Interference Pattern

We assume the following scheme of PS and PD of ILS: the sources of laser radiation, located, respectively, at points $\left\{0,\;\rho_{1}\right\}$ and $\left\{0,\;\rho_{2}\right\}$, emit collimated laser beams parallel to each other and to the *OX*-axis in the direction of positive *x* values; and PD is located at point *Q* (Figure 1) with coordinates {x, ρ}. We adopt that the quantity $\rho_{tr}$ means the vector of spacing of radiative sources, producing the interference pattern. Because the two laser beams that are used to form the interference pattern are obtained by splitting one initial laser beam (see Figure 1), in the initial plane *x* = 0 the function of second-order mutual coherence of the field of each of these laser beams, representing the Gaussian partially coherent beam, has the following form:

(1) $$\Gamma_{2}\left(x\;=\;0,\;\rho^{\prime },\;\rho^{\prime \prime };\;\rho_{j},\;\rho_{j}\right)\;=\;E_{0}^{2}\;\exp\left\{\;-\left[{\left(\rho^{\prime }\;-\;\rho_{j}\right)}^{2}\;+\;{\left(\rho^{\prime \prime }\;-\;\rho_{j}\right)}^{2}\right]/\left(2\;a_{0}^{2}\right)\;-\;{\left(\rho^{\prime }\;-\;\rho^{\prime \prime }\right)}^{2}/\rho_{k}^{2}\right\}$$

where $E_{0}$ is the initial amplitude at the optical beam axis; $a_{0}$ is the initial beam radius; $\rho_{k}$ is the spatial coherence radius of the initial field; k = 2 π/λ, λ is the wavelength of the optical radiation in vacuum; $\rho^{\prime }$, $\rho^{\prime \prime }$ are the position vectors of the observation points; j = 1, 2. A similar formula also holds for second-order mutual coherence function of the fields of these two laser beams:

(2) $$\begin{array}{l} {\tilde{\Gamma }}_{2}\left(x\;=\;0,\;\rho^{\prime },\;\rho^{\prime \prime };\;\rho_{j},\;\rho_{j^{\prime }}\right)\;\\ =\;E_{0}^{2}\;\exp\left\{-\left[{\left(\rho^{\prime }\;-\;\rho_{j}\right)}^{2}\;+\;{\left(\rho^{\prime \prime }\;-\;\rho_{j^{\prime }}\right)}^{2}\right]/\left(2\;a_{0}^{2}\right)\;-\;{\left[\left(\rho^{\prime }\;-\;\rho_{j}\right)\;-\;\left(\rho^{\prime \prime }\;-\;\rho_{j^{\prime }}\right)\right]}^{2}/\rho_{k}^{2}\right\} \end{array}$$

where $j\;\neq \;j^{\prime }$, and $j,\;j^{\prime }\;=\;1,\;2$. We consider that, in the formation of the second radiative source, it is admissible to assume that its coherence properties are totally identical to the initial source and are just shifted in space by the amount $\rho_{tr}$.

PD is the square-law detector that responds to the power of incoming radiation, the signal of which can be represented as $i_{r}\left(x,\;\rho \right)\;=\;\eta_{0}\;I\left(x,\;\rho \right)$, where $\eta_{0}$ is the quantum efficiency coefficient of PD; I(x, ρ) is the instantaneous value of intensity of interference pattern at the detector location, which has the form:

(3) I(x, ρ) = U1(x, ρ) U1∗(x, ρ) + U2(x, ρ) U2∗(x, ρ) + 2Re[U1(x, ρ) U2∗(x, ρ)]

where $U_{j}\left(x,\;\rho \right)$ is the field of optical wave of one source; j = 1, 2. Suppose that $\rho_{1}\;=\;-\;\rho_{tr}/2$, and $\rho_{2}\;=\;\rho_{tr}/2$.

The second-order mutual coherence function of fields of two partially coherent Gaussian beams of optical radiation for boundary Equations (1) and (2), written with the use of the extended Huygens-Fresnel principle in the case of square-law approximation for the function, which describes the effect of random inhomogeneities of the medium, has the following form [4]:

(4) $$\langle U_{j}\left(x,\;\rho \right)\;U_{j^{\prime }}^{*}\left(x,\;\rho \right)\rangle \;=\;\frac{E_{0}^{2}\;a_{0}^{2}}{a^{2}\left(x\right)}\;\exp[-\;\frac{{\left(\rho \;-\;\rho_{j}\right)}^{2}}{2\;a^{2}\left(x\right)}\;-\;\frac{{\left(\rho \;-\;\rho_{j^{\prime }}\right)}^{2}}{2\;a^{2}\left(x\right)}\;-\;i\;\frac{\delta \left(x\right)}{a^{2}\left(x\right)}\;\left(\rho_{j}\;-\;\rho_{j^{\prime }}\right)\;\rho \;+\;i\;\frac{\delta \left(x\right)}{2\;a^{2}\left(x\right)}\;\left(\rho_{j}^{2}\;-\;\rho_{j^{\prime }}^{2}\right)\;-\;\frac{{\left(\rho_{j}\;-\;\rho_{j^{\prime }}\right)}^{2}}{\rho_{c}^{2}\left(x\right)}]$$

where $a\left(x\right)\;=\;a_{0}\;{\left[1\;+\;\Omega_{0}^{-2}\;\left(1\;+\;a_{0}^{2}/\rho_{k}^{2}\;+\;\frac{4}{3}\;a_{0}^{2}/\rho_{0}^{2}\right)\right]}^{1/2}$ is the mean radius of the laser beam; $\delta \left(x\right)\;=\;\Omega_{0}^{-1}\;\left(1\;+\;a_{0}^{2}/\rho_{k}^{2}\right)$ is the geometrical factor; $\delta \left(x\right)/\left[k\;a^{2}\left(x\right)\right]$ is the difference between curvatures of laser beam wavefronts; $\rho_{c}\left(x\right)\;=\;3^{1/2}\;\rho_{0}\;{\left\{\left[1\;+\;\Omega_{0}^{-2}\;\left(1\;+\;\frac{a_{0}^{2}}{\rho_{k}^{2}}\;+\;\frac{4}{3}\;\frac{a_{0}^{2}}{\rho_{0}^{2}}\right)\right]/\left[\Omega_{0}^{-2}\;\left(1\;+\;\frac{a_{0}^{2}}{\rho_{k}^{2}}\right)\;+\;\frac{3}{4}\;\frac{\rho_{0}^{2}}{\rho_{k}^{2}}\right]\right\}}^{1/2}$ is the radius of mutual coherence of partially coherent laser beams; $\Omega_{0}\;=\;k\;a_{0}^{2}/x$ is the Fresnel number of emitting aperture; $\rho_{0}\;=\;{\left(0.3642\;C_{\varepsilon }^{2}\;k^{2}\;x\right)}^{-3/5}$ is the coherence radius of plane optical wave in the turbulent atmosphere; $C_{\varepsilon }^{2}$ is the structure parameter of atmospheric turbulence; and $j,\;j^{\prime }\;=\;1,\;2$.

Using Equations (3) and (4), we obtain the formula for the mean value of intensity of interference pattern:

(5) $$\langle I\left(x,\;\rho \right)\rangle \;=\;2\;\frac{E_{0}^{2}\;a_{0}^{2}}{a^{2}\left(x\right)}\;\exp\left[-\;\frac{\rho^{2}\;+\;\rho_{tr}^{2}/4}{a^{2}\left(x\right)}\right]\;\left\{\cosh\left[\frac{\rho_{tr}\;\rho }{a^{2}\left(x\right)}\right]\;+\;\exp\left[-\;\frac{\rho_{tr}^{2}}{\rho_{c}^{2}\left(x\right)}\right]\;\cos\left[\frac{\delta \left(x\right)}{a^{2}\left(x\right)}\;\rho_{tr}\;\rho \right]\right\}$$

Equation (5) can be used to formulate the conditions, restricting the choice of parameters of laser beams and ILS scheme. The linear dimensions $l_{\mathrm{int}}\left(x\right)$ of the region, where the interference pattern Equation (5) had formed, provided that $a\left(x\right)\;\rangle \rangle \;\rho_{tr}$, have a value approximately equaling the current laser beam diameter:

(6) $$l_{\mathrm{int}}\left(x\right)\;≅\;2\;a\left(x\right)$$

The band maxima of interference pattern are at the points $\rho_{\max}$, determined (see Equation (5)) from equation of the form: $\delta \left(x\right)/a^{2}\left(x\right)\;\rho_{tr}\;\rho_{\max}\;=\;2\;n\;\pi$, where n = 0, ± 1, ±2, …; while the band minima are at points $\rho_{\min}$ determined from $\delta \left(x\right)/a^{2}\left(x\right)\;\rho_{tr}\;\rho_{\min}\;=\;\left(2\;n\;+\;1\right)\;\pi$, n = 0, ± 1, ±2, …. Let $\rho \;\|\;\rho_{tr}$, then $\rho_{\max}\;\|\;\rho_{\min}\;\|\;\rho_{tr}$ and $\rho_{\max}\;=\;2\;n\;\pi \;a^{2}\left(x\right)/\left[\delta \left(x\right)\;\rho_{tr}\right]$, $\rho_{\min}\;=\;\left(2\;n\;+\;1\right)\;\pi \;a^{2}\left(x\right)/\left[\delta \left(x\right)\;\rho_{tr}\right]$, while the width of the interference band $\Delta l_{\mathrm{int}}\left(x\right)$ can be evaluated from the following formula:

(7) $$\Delta l_{\mathrm{int}}\left(x\right)\;=\;2\;\left|\rho_{\max}\;-\;\rho_{\min}\right|\;=\;2\;\pi \;a^{2}\left(x\right)/\left[\delta \left(x\right)\;\rho_{tr}\right]$$

On the other hand, the contrast of distorted interference pattern, determined from the mean intensity (at $\rho_{\max}\;\approx \;\rho_{\min}\;\approx \;\rho$), is equal to:

(8) $$\nu \;=\;\frac{\langle I\left(x,\;\rho_{\max}\right)\rangle \;-\;\langle I\left(x,\;\rho_{\min}\right)\rangle }{\langle I\left(x,\;\rho_{\max}\right)\rangle \;+\;\langle I\left(x,\;\rho_{\min}\right)\rangle }\;≅\;{\left\{\cosh\left[\frac{\rho_{tr}\;\rho }{a^{2}\left(x\right)}\right]\right\}}^{-1}\;\exp\left[-\;\frac{\rho_{tr}^{2}}{\rho_{c}^{2}\left(x\right)}\right]$$

It is evident that, in order for ILS to be operable, at least one complete interference band should be in the field of interference pattern; therefore, Equations (6) and (7) make it possible to formulate the condition: $l_{\mathrm{int}}\left(x\right)\;\geq \;\Delta l_{\mathrm{int}}\left(x\right),$ which is fulfilled when the range between the sources of laser radiation satisfies the condition:

(9) $$\rho_{tr}\;\geq \;\pi \;a\left(x\right)/\delta \left(x\right)$$

Moreover, the contrast of the mean interference pattern near its center (when $\cosh\left[\rho_{tr}\;\rho /a^{2}\left(x\right)\right]≅\;1$) will be satisfactory until the coherence radius of optical field at the observation point exceeds the value of transversal shift of the beams; therefore, from Equation (8) we obtain the following inequality:

(10) $$\rho_{tr}\;\leq \;\sqrt{-\;\ln\left(\nu \right)}\;\rho_{c}\left(x\right)$$

Summing two conditions, Equations (9) and (10), we obtain the formula of the following form:

(11) $$\pi \;a\left(x\right)/\delta \left(x\right)\;\leq \;\rho_{tr}\;\leq \;\sqrt{-\;\ln\left(\nu \right)}\;\rho_{c}\left(x\right)$$

In order for Equation (11) to be fulfilled, the right-hand side of the inequality should exceed the left-hand side, making it possible to formulate the condition restricting the initial sizes of the laser beams $a_{0}$:

(12) $$a_{0}\;\leq \;\frac{\sqrt{-\;\ln\left(\nu \right)}}{\pi }\;\left(1\;+\;\frac{a_{0}^{2}}{\rho_{k}^{2}}\right)/\sqrt{\frac{1}{3\;\rho_{0}^{2}}\;\left(1\;+\;\frac{a_{0}^{2}}{\rho_{k}^{2}}\right)\;+\;\frac{1}{4\;\rho_{k}^{2}}\;\frac{k^{2}\;a_{0}^{4}}{x^{2}}}$$

## 3. Results

### 3.1. Choice of Parameters of ILS Optical Scheme

**Figure 2:**
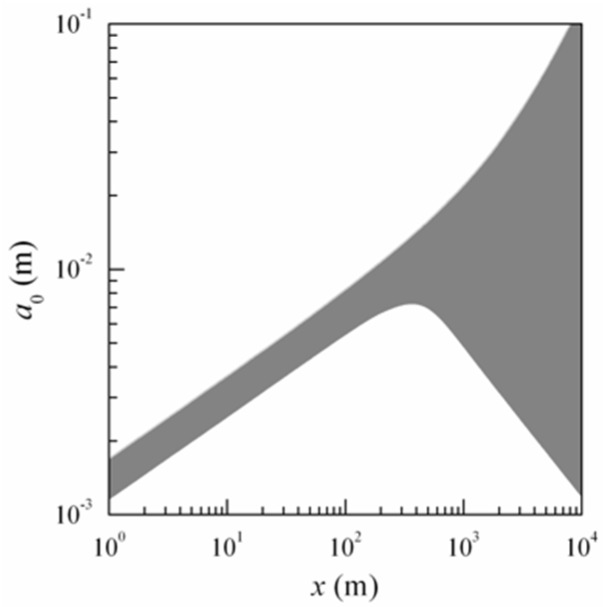
The nomogram for choosing the initial laser beam radii $a_{0}$.

Figure 2 presents the behavior of implicitly specified function $a_{0}\left(x\right)$, defined by Equation (12). Values of $a_{0}$, lying on the curve that graphically represents the solution of Equation (12), correspond to the condition “=” in Equation (12). The $a_{0}$ values, lying below the corresponding curve, correspond to the condition “〈” in Equation (12). All curves in Figure 2, plotted for different parameters of the problem, λ, $\rho_{k}$, $C_{\varepsilon }^{2}$, and ν, are within grey-colored region. Figure 2 shows an assemblage of curves in the form of a nomogram for the initial laser beam radii $a_{0}$. This figure shows two regions (not filled) that allow formation of a stable interference pattern. In filled area (grey) is not possible formation of a stable pattern. The effect of the wavelength of optical radiation λ on the value of the initial size of laser beams $a_{0}$ was estimated by considering the values of λ in the range from 0.51 μm to 1.55 μm (at the same time, the other parameters were as follows: $\rho_{k}\;=$ 2 cm, ν = 0.1, $C_{\varepsilon }^{2}\;=$ 10^−13^ m^−2/3^). Correspondingly, the contrast of the interference pattern ν varied from 0.1 to 0.5 (at λ= 1.55 μm, $\rho_{k}\;=$ 2 cm, $C_{\varepsilon }^{2}\;=$ 10^−13^ m^−2/3^); and the structure parameter of the atmospheric turbulence $C_{\varepsilon }^{2}$ varied from 10^−16^ m^−2/3^ to 10^−13^ m^−2/3^ (at λ= 1.55 μm, $\rho_{k}\;=$ 2 cm, ν = 0.1).

Based on data presented in Figure 2, the initial laser beam radii $a_{0}$ can be chosen to be 1…2 mm for pathlengths in the range from 1 m to 10 km. Equation (12) must be solved (using the condition “=”) with respect to $\rho_{k}$ to determine the minimum acceptable level of the spatial coherence radius of initial field. 

**Figure 3:**
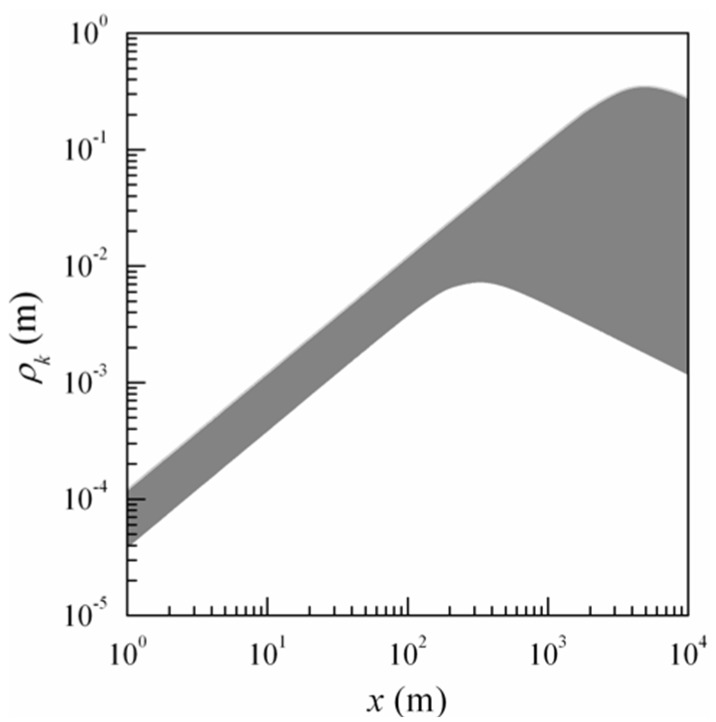
The nomogram for choosing the spatial coherence radius of the initial field of laser beams $\rho_{k}$.

The data thus obtained are presented in Figure 3 in the form of grey-colored region (in these estimates, the contrast of interference pattern ν varied from 0.1 to 0.5, and the structure parameter of atmospheric turbulence $C_{\varepsilon }^{2}$ varied from 10^−16^ m^−2/3^ to 10^−13^ m^−2/3^); and any curve, obtained in solution of Equation (12), falls within this region.

The shape and position of the grey-colored region, presented in Figure 3, indicate that the initial coherence of optical sources plays the key role (in selecting the parameters of single beams) along propagation paths up to 300 m in length, while atmospheric turbulence is critical when *x* > 300 m. Figure 3 shows an assemblage of curves in the form of a nomogram for choosing the spatial coherence radius $\rho_{k}$ of the initial field of laser beams that allow the formation of a stable interference pattern. Here also, as in the previous figure, showing the two areas (not filled) that allow formation of a stable interference pattern. Data in Figure 3 demonstrate that the laser beam coherence level, comparable to the initial coherence radius of 1…2 cm, is reached on the paths with the lengths *x* ≥ 300 m. Thus, it can be further assumed that $\rho_{k}\;=$ 1…2 cm. For laser beams with these parameters, the angular beam width $\psi_{0}$ in the region, where there already exists the directional diagram equaling $\psi_{0}\;≅\;2/\left(k\;a_{0}\right)\;{\left(1\;+\;a_{0}^{2}/\rho_{k}^{2}\right)}^{1/2}$, must not exceed ≈ 1’.

**Figure 4:**
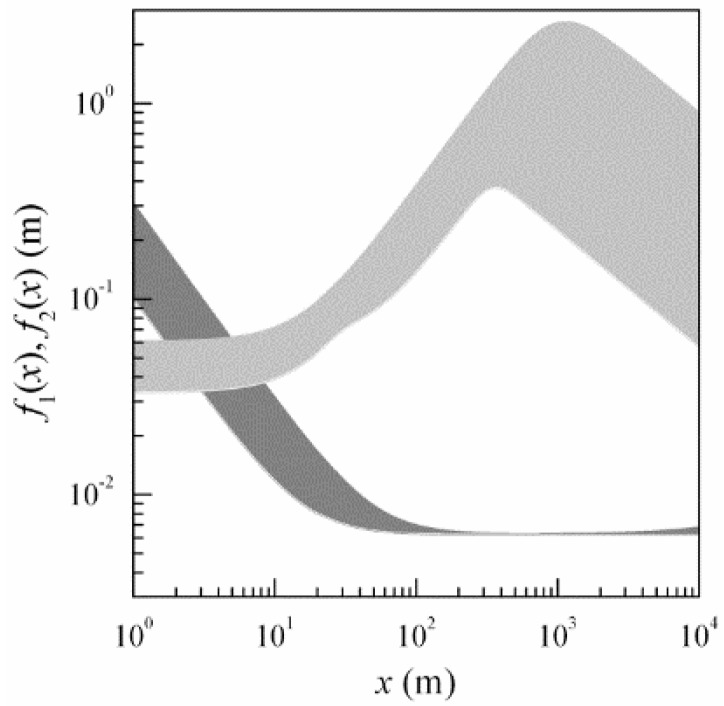
The nomogram for choosing the spacing between optical axes of laser beams $\rho_{tr}$. The grey-colored region shows the variability range of the function $f_{1}\left(x\right)$ , and the region in light grey depicts the variability range of the function $f_{2}\left(x\right)$ .

Euqation (11) for known parameters of the optical beams makes it possible to choose the distance between radiative sources $\rho_{tr}$. Figure 4 presents the behavior of the functions:

(13) $$f_{1}\left(x\right)\;=\;\pi \;\frac{k\;a_{0}^{3}}{x}\;\sqrt{1\;+\;\frac{x^{2}}{k^{2}\;a_{0}^{4}}\;\left(1\;+\;\frac{a_{0}^{2}}{\rho_{k}^{2}}\;+\;\frac{4}{3}\;\frac{a_{0}^{2}}{\rho_{0}^{2}}\right)}/\left(1\;+\;\frac{a_{0}^{2}}{\rho_{k}^{2}}\right)$$

(grey-colored region) and

(14) $$f_{2}\left(x\right)\;=\;\sqrt{-\;\ln\left(\nu \right)}\;\sqrt{\left[1\;+\;\frac{x^{2}}{k^{2}\;a_{0}^{4}}\;\left(1\;+\;\frac{a_{0}^{2}}{\rho_{k}^{2}}\;+\;\frac{4}{3}\;\frac{a_{0}^{2}}{\rho_{0}^{2}}\right)\right]/\left[\frac{1}{3\;\rho_{0}^{2}}\;\frac{x^{2}}{k^{2}\;a_{0}^{4}}\;\left(1\;+\;\frac{a_{0}^{2}}{\rho_{k}^{2}}\right)\;+\;\frac{1}{4\;\rho_{k}^{2}}\right]}$$

(region in light grey), calculated for different parameter values of the problem. Figure 4 shows nomograms for choosing the values $\rho_{tr}$, at which the formation of interference pattern is a stable. 

In estimating the dimensions of both regions, presented in Figure 4, we quantified both the effect of wavelength of the optical radiation λ (λ in the range from 0.51 μm to 1.55 μm for $a_{0}\;=$ 2 mm, $\rho_{k}\;=$ 2 cm, ν = 0.1, $C_{\varepsilon }^{2}\;=$ 10^−16^ m^−2/3^), and the effect of the contrast of interference pattern ν (ν in the range from 0.1 to 0.5 for λ = 1.55 μm, $a_{0}\;=$ 2 mm, $\rho_{k}\;=$ 2 cm, $C_{\varepsilon }^{2}\;=$ 10^−16^ m^−2/3^), as well as the effect of structure parameter of atmospheric turbulence $C_{\varepsilon }^{2}$ ($C_{\varepsilon }^{2}$ in the range from 10^−16^ m^−2/3^ to 10^−14^ m^−2/3^ for λ = 1.55 μm, $a_{0}\;=$ 2 mm, $\rho_{k}\;=$ 2 cm, ν = 0.1).

Considering that the spacing $\rho_{tr}$ of radiative sources that produce interference pattern must be larger than the values of the function $f_{1}\left(x\right)$ and smaller than the values of the function $f_{2}\left(x\right)$, data of Figure 4 for pathlengths *x* from 20 m to 10 km can be used to estimate the admissible values of $\rho_{tr}$ in the range from 1 cm to 5 cm. It is just these spacings of radiative sources $\rho_{tr}$ that were used below to estimate ILS operability.

**Figure 5:**
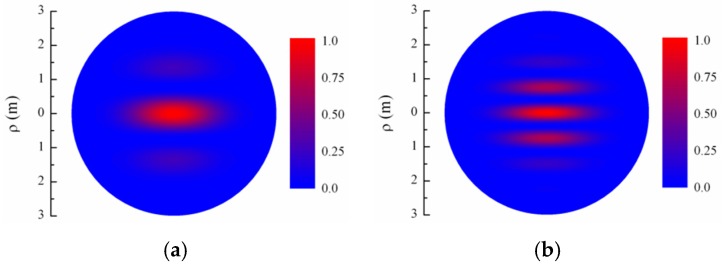

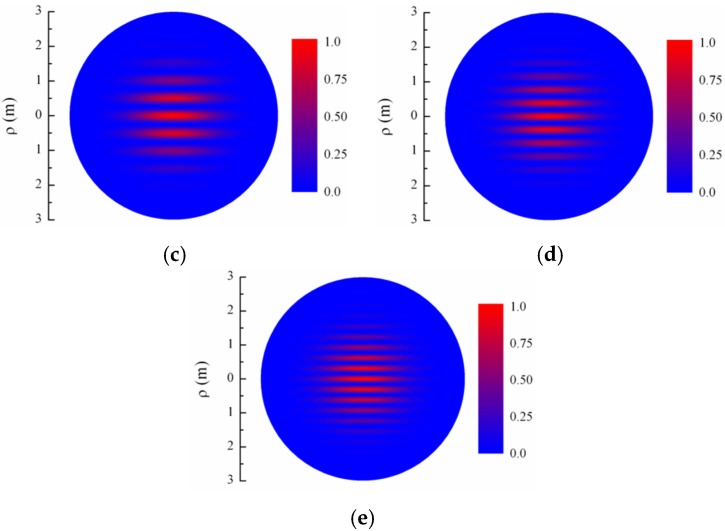
The average intensity of interferometric laser scanning (ILS) interference pattern for different values of $\rho_{tr}$: (**a**) 1 cm; (**b**) 2 cm; (**c**) 3 cm; (**d**) 4 cm; and (**e**) 5 cm.

Figure 5 presents the calculations of the average intensity of ILS interference pattern (Figure 1) at $C_{\varepsilon }^{2}\;=$ 10^−16^ m^−2/3^ and x= 10 km for different distances between centers of two identical collimated Gaussian partially coherent beams (λ = 1.55 μm, $a_{0}\;=$ 2 mm, $\rho_{k}\;=$ 2 cm): (a) $\rho_{tr}\;=$ 1 cm, (b) $\rho_{tr}\;=$ 2 cm, (c) $\rho_{tr}\;=$ 3 cm, (d) $\rho_{tr}\;=$ 4 cm, and (e) $\rho_{tr}\;=$ 5 cm. Panels in Figure 5a–e represent the color polar contour plots (100 shades of two colors).

Figure 5 shows the five options of the interference pattern where the method is workable in principle. The figure illustrates the real (calculated) interference pattern and that it represents. The Figure 5 corresponds to the high visibility at the weak turbulence and support the method performance:

1. For $\rho_{tr}\;=$ 1 cm, the interference pattern starts to form at separation of 30 m from the sources of the optical radiation, and becomes finally formed at the range of 300 m, comprising three bright bands, with central band becoming most intense starting from 40 m, and with two sideward bands, located symmetrically about the central band, being identical.
2. For $\rho_{tr}\;=$ 2 cm, the interference pattern starts to form at the range of 30 m from the sources of optical radiation, and becomes finally formed at the range of 300 m. In this case, the pattern has seven intense bands; the central band becomes most intense starting from 80 m; and sideward bands are pairwise identical.
3. For $\rho_{tr}\;=$ 3 cm, the interference pattern starts to form at the range of 30 m from the sources of the optical radiation, and becomes ultimately formed at the range of 300 m, comprising 11 intense bands, with the central band becoming the brightest starting from 120 m.
4. For $\rho_{tr}\;=$ 4 cm, the interference pattern starts to form at the range of 40 m from the sources of the optical radiation, and becomes ultimately formed at the range of 400 m, comprising 15 intense bands, with the central band becoming most intense starting from 170 m.
5. For $\rho_{tr}\;=$ 5 cm, the interference pattern starts to form at the range of 40 m from the sources of the optical radiation, and becomes ultimately formed at the range of 500 m, comprising 19 intense bands, with central band becoming most intense starting from 250 m.

### 3.2. Contrast of ILS Interference Pattern in the Turbulent Atmosphere

**Figure 6:**
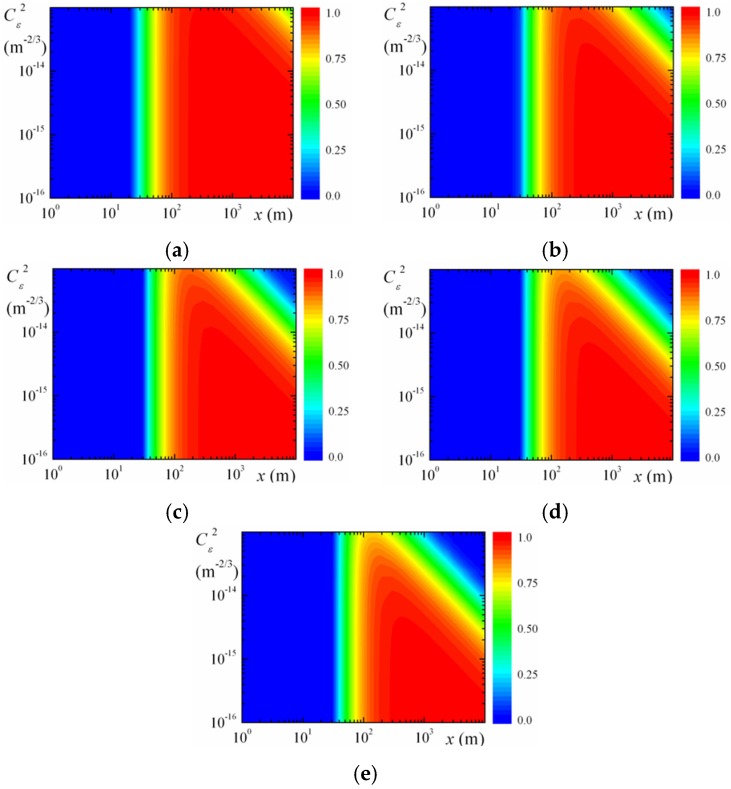
The contrast of ILS interference pattern for different distances between the centers of emitting beam apertures $\rho_{tr}$: (**a**) 1 cm; (**b**) 2 cm; (**c**) 3 cm; (**d**) 4 cm; and (**e**) 5 cm.

The contrast of ILS interference pattern can be estimated according to approximate formula, obtained from Equation (8). Figure 6 presents estimates of the contrast of ILS interference pattern (Figure 1) for all values of structure parameter of atmospheric turbulence, realizable in near-water atmospheric layer [5,6]. Figure 6 gives estimates of the visibility of the interference patterns at different levels of turbulence. It was assumed that laser radiation propagated along horizontal path at height of 10…20 m above water surface. The color (40 colors) contour plots, presented in Figure 6a–e, demonstrate the dependence of the contrast of ILS interference pattern (ν) on the laser beam propagation path length (*x*) and on the value of the structure parameter of atmospheric turbulence ($C_{\varepsilon }^{2}$) at the wavelength of the optical radiation λ = 1.55 μm for different ranges between the centers of two identical collimated Gaussian partially coherent beams ($a_{0}\;=$ 2 mm, $\rho_{k}\;=$ 2 cm): (a) $\rho_{tr}\;=$ 1 cm, (b) $\rho_{tr}\;=$ 2 cm, (c) $\rho_{tr}\;=$ 3 cm, (d) $\rho_{tr}\;=$ 4 cm, and (e) $\rho_{tr}\;=$ 5 cm.

The red-colored regions in Figure 6a–e show the laser beam propagation path lengths *x* for different structure parameters of the atmospheric turbulence $C_{\varepsilon }^{2}$, when high contrasts of ILS interference pattern in the turbulent atmosphere take place

## 4. Conclusions

At higher contrasts of interference pattern, ILS proves to be operable in a narrower range of spacings of the sources of optical radiation that produce the interference pattern. The closer the sources of optical radiation to each other, the lower the contrast of the recorded interference pattern, and, at last, the longer the wavelength of the optical radiation, the greater is ILS operability region. For the wavelength of the optical radiation λ = 1.55 μm, at spacings of the sources $\rho_{tr}$ from 1 cm to 5 cm under the conditions of the mean turbulence intensity value of 10^−15^ m^−2/3^, most often realized in the maritime and coastal atmosphere at midlatitudes, the operability range of the device may reach 10 km and longer, depending on the geometric positions of optical elements.

## Abbreviations

The following abbreviations are used in this manuscript:

ILS: Interferometric laser scanning
PS: Piezoelectric scanner
EOM: Electro-optic modulator
SFG: Sweep-frequency generator
PD: Photodetector
BFD: Beam forming device
